# Predicting Viable Skin Concentration: Modelling the Subpapillary Plexus

**DOI:** 10.1007/s11095-022-03215-z

**Published:** 2022-03-09

**Authors:** Joshua J. Calcutt, Michael S. Roberts, Yuri G. Anissimov

**Affiliations:** 1 School of Environment and Science, Griffith University, Gold Coast, Queensland 4222 Australia; 2 University of South Australia, Clinical Health Sciences and Basil Hetzel Institute for Translational Health Research, Adelaide, 5011 Australia; 3 Diamantina Institute, University of Queensland, Brisbane, 4072 Australia

**Keywords:** Viable skin concentration, Subpapillary plexus, Computational modelling, COMSOL

## Abstract

The skin concentration of a substance after a topical application or exposure determines both local treatment outcomes and the dermal toxicity assessment of various products. However, quantifying the time course of those concentrations at skin effect sites, such as the viable epidermal, superficial dermis and appendages in humans is especially problematic in vivo, making physiologically based mathematical modelling an essential tool to meet this need. This work further develops our published physiologically based pharmacokinetic and COMSOL based dermal transport modelling by considering the impact of the superficial subpapillary dermal plexus, which we represent as two well stirred compartments. The work also studied the impact on dermal concentrations of subpapillary plexus size, depth, blood velocity and density of subpapillary plexus vessels. Sensitivity analyses are used to define the most important transport determinants of skin concentrations after topical application of a substance, with previously published results used to validate the resulting analyses. This resulting model describes the available experimental data better than previous models, especially at deeper dermal depths.

## Introduction

Human skin exposure to various substances, whether accidental (as in case of herbicides and pesticides) or intentional (as in cosmetic or dermatological applications), may lead to systemic or local dermal toxicity. While systemic toxicity in this case is determined by the flux of the substance though the skin into the systemic circulation, and is relatively well studied, the skin toxicity after topical application depends on the substance concentration at reactive skin sites. This concentration, in turn, is dependent on the flux of solute through the stratum corneum, clearance from those sites and, where the target site is the superficial dermis, drug transport in the dermal tissues (1–6).

In our earlier work, we represented transport in the dermis in a physiologically based pharmacokinetic model whereby a substance was carried to deeper tissues via an axial convective-diffusion process and cleared by radial convective process, whereby convection consists of transport by blood flow, lymphatics and induced fluid flow (3, 4). This work showed that convection could dominate axial dermal and deeper tissue transport for the highly plasma protein bound corticosteroids (3) and non-steroidal anti-inflammatory drugs (4). However, dermal capillary loops should also be considered in optimising viable skin modelling as we and others have shown using capillary models (1, 2, 7). In particular, we found that convectional transport in the dermal capillaries has a profound effect on the time course of dermal drug concentrations, including the time taken to reach a steady state concentration (1).

In this paper, we applied our previously published COMSOL based detailed computational models (1, 2) to develop the more sophisticated and realistic physiological representation of dermal transport described in this paper. Here, we refine the modelling of the dermal capillary network to account for a highly anastomotic network of blood vessels in the superficial dermis, the subpapillary plexus. Since explicitly modelling highly interconnected capillaries represents a significant computational challenge, including with COMSOL, we used a simplified representation of this complex network by representing the arteriole plexus and venous plexus as two separate well stirred compartments. Physiological parameters, such as the distance between capillary loops, depth of the capillary loop, capillary loop width and velocity and permeability of the capillary loops described in our previous work (1, 2) were also used in the present model.

The new capillary model developed provides a more realistic representation of drug transport in the viable skin and blood vessels which is important for cases when significant amount of drug can permeate to deeper skin layers. The complexity of the new model is required to take into account convective transport in the capillary network of the viable skin. It is especially important when the site of action for a topically administered product are the deeper tissues below the skin, as applies for certain anti-inflammatory medications and when the skin delivery techniques bypass the epidermis. In each case, a quality-by-design approach to topical product design requires the prediction of a safe and effective concentration be delivered at that site (6).

## Methods

### Skin Transport Model

**Fig. 1:**
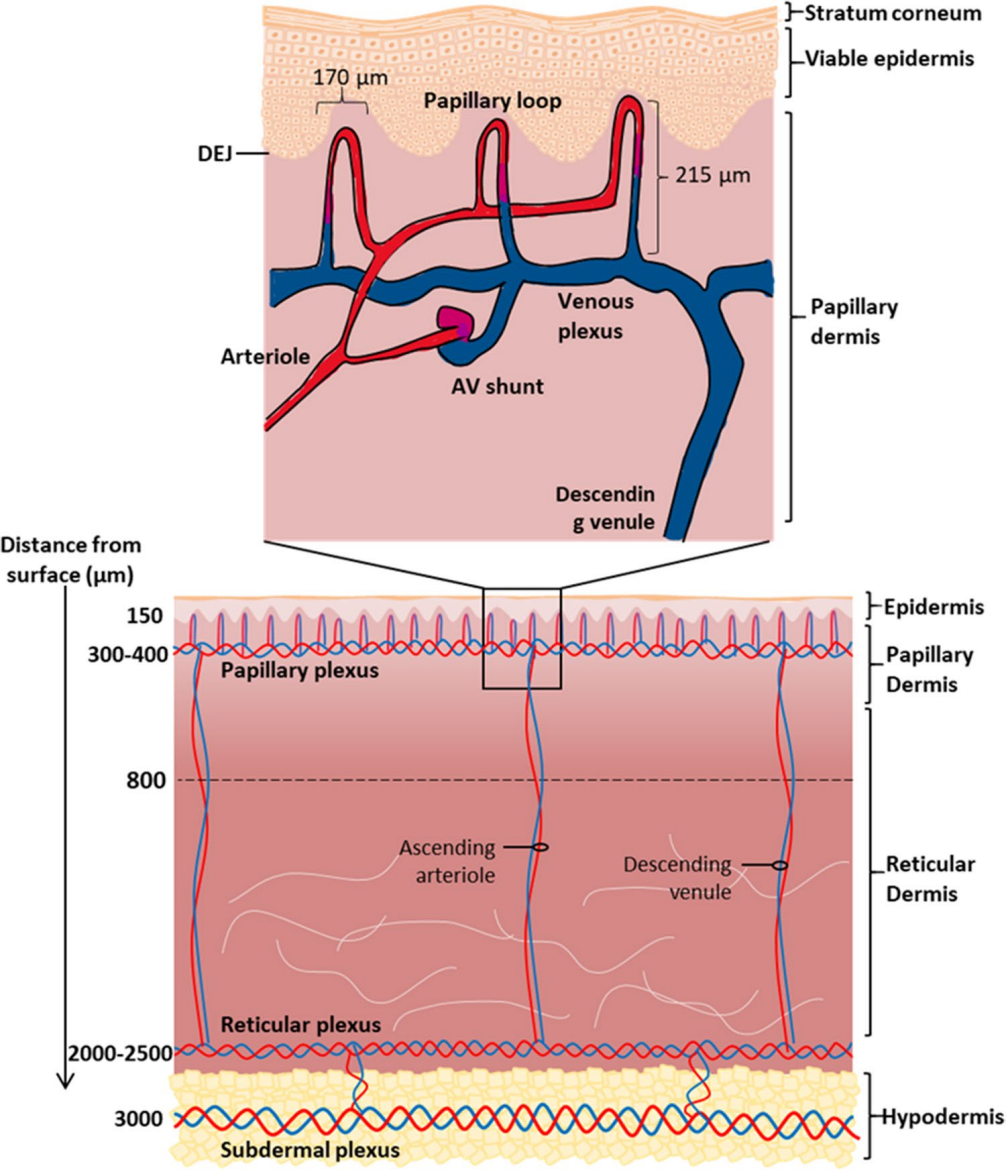
Schematic of blood circulation in the skin

Figure 1 shows both capillary loops and arteriovenous anastomoses (AVAs or AV shunts) in the dermis. Whilst capillary loops continually move blood backwards and forwards though the tissue, AV shunts bypass the tissues in the thermoregulation of the skin. As AVAs are mainly limited in distribution to the body extremities and closed when the ambient temperature is below the lower end of the thermoneutral zone (8), they are not discussed further in any more detail in this paper. However, it is also recognised that, when the body temperature is at the upper end of the thermoneutral zone when AVAs will be most likely open, they can impact on dermal drug disposition (8).

**Fig. 2:**
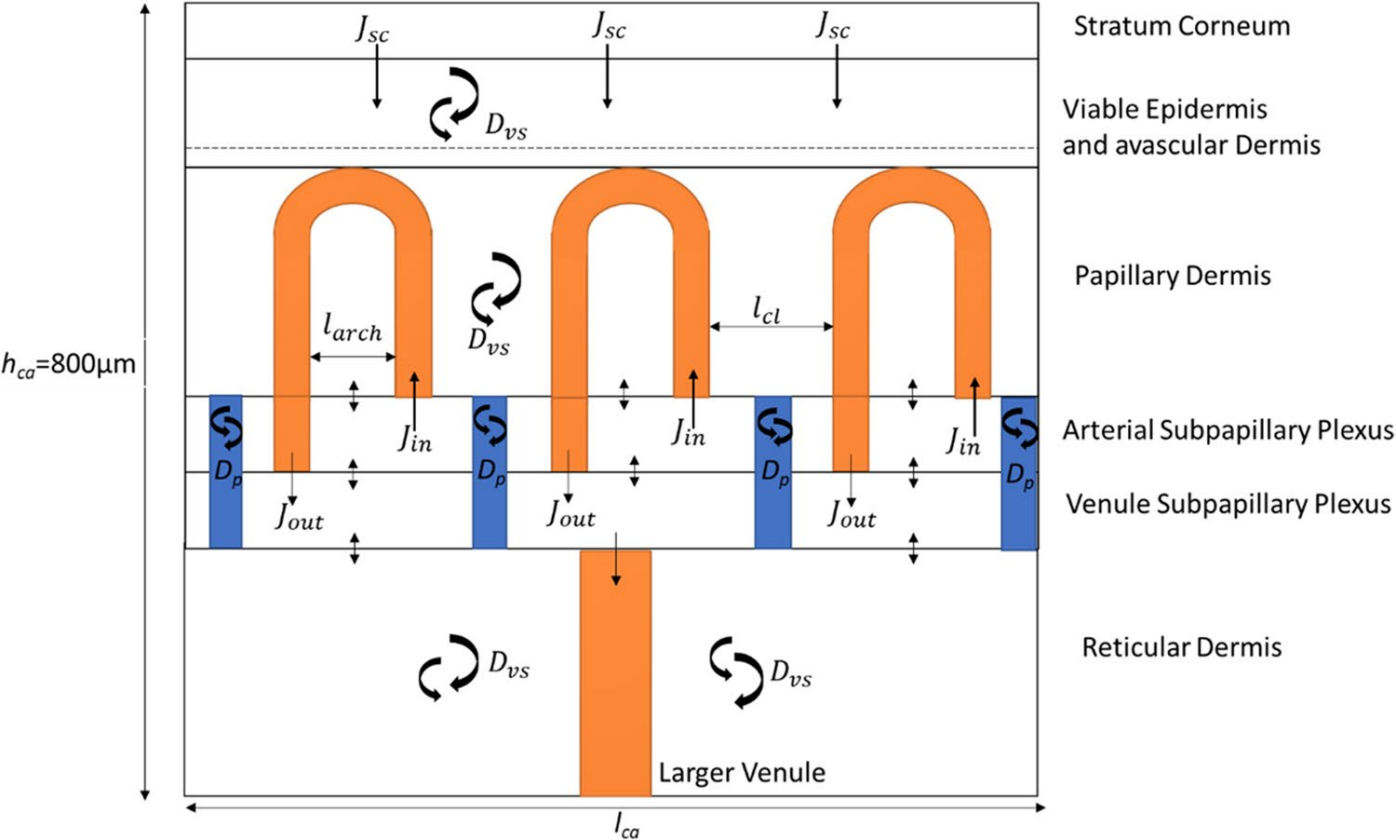
An illustration for the composition of the model developed for the subpapillary plexus. The *l*_*ca*_ represents the length of the computational area, *h*_*ca*_ represents the thickness, *J*_*in*_ is the flux of drug entering the capillary loops from the arteriole plexus, *J*_*out*_ is the flux of drug entering the venule plexus from the capillary loop, *D*_*p*_ represents the diffusion coefficient between the venules and arterioles in the plexus and *D*_*vs*_ is the diffusion coefficient in the viable skin

Figure 2 shows the skin transport model used in this work. Transport is assumed to be defined by both the 3-D geometry of the superficial (papillary) dermis and the various physiological parameters defining transport in an improved viable skin transport model. Within the new model, the depth of the capillary loops below the epidermal-dermal junction was set at 150μm. The capillary loop width was defined as 170μm, based on the experimental data reported by Braverman (9). An estimated distance between each capillary loop of 70μm (10) and a superficial dermis depth of 800μm (defining the depth of the considered slab of skin) were used to avoid complicating the model by the further inclusion of the much larger subcutaneous plexus, which lies below this depth (11).

This model, in recognising the importance of dermal blood flow as a clearance mechanism, incorporates the velocity and permeability of the capillary loops as important physiological parameters. Both of these factors were recognised in our previous capillary model which looked explicitly at convective transport in the capillary loops (1). According to Fragrell et al. (12), the velocity of the capillary loop’s blood is 0.65 ± 0.3 *mm*/*s*. Meanwhile, Krestos et al. estimated that the permeability of the endothelial lining of the capillary loops was 1 × 10^−6^*m*/*s* (13).

The model also recognises that both the capillary loops and the subpapillary plexus below the loops may modify the dermal transport of substances. The subpapillary plexus is composed of multiple blood vessels that can be broken into terminal arterioles, venous and arterial capillaries, and postcapillary venules (11). Here, we used the length of the capillary loop to define the depth of the plexus. Previous studies have reported that these capillary loops connect to the subpapillary plexus, with the length of the capillary loop being 215μm with a standard deviation of 40μm. (14). As a consequence, the depth of the subpapillary plexus was set at 300-400μm and matched the findings of (15).

The subpapillary plexus is predominately composed of postcapillary venules and venous and arteriole capillaries. Each of the vessels mentioned above has a larger diameter than the capillary loops. The postcapillary venules have a diameter of 18-23μm. The venous capillaries have a diameter of 12-35μm, while the arteriole diameter is 17-26μm (11, 16). The subpapillary plexus size was determined from these diameter values as it was assumed there was one venous capillary with a maximum diameter and some branching capillaries in the subpapillary plexus. By using this assumption, the depth of the total plexus could be assumed to be 50μm. As our model splits the plexus into two layers, the arteriole and venular plexus and each layer was assumed to be the same size of 25 *μ*m. However, both of these variables will be changed in the sensitivity analysis to see the impact on transport.

The subpapillary plexus is also sourced by arteries that travel vertically through the reticular dermis. Similarly, there are venules in the reticular dermis that transport drug vertically to the subcutaneous plexus. In this model, the carry down effect of the vessels (i.e. axial as distinct from radial transport with respect to the surface) is a central research question. For this reason, the model recognises that these vertical venules as extensions to the venular branch of the capillary loop, recognising that this extension of the capillary loops is not seen in previous capillaroscopy studies but that this model is a simplified representation of deeper vasculature systems (11, 17) and complex plexus capillary branching. The capillaries differ from the larger venules in density, size, morphology and permeability (17). Importantly, the model matches the vascularization of the human thumb as described by Geyer et al. (18) and has an appropriate geometry complexity to undertake the computations envisaged here.

### Drug Transport Equations

The governing equations that describe drug transport in the subpapillary plexus of the skin in this model are an extension of those described in our previous model (1). In brief, the equations that describe drug transport in the skin have been developed as follows. In the upper regions of the avascular viable epidermis, where there are no capillaries or lymphatic vessels, convection is unlikely and diffusion can be assumed to be the main mechanism for drug transport.

A corresponding three-dimensional drug transport expression for transport in the viable epidermis is then:

$$\frac{\partial {C}_{vs}}{\partial t}={D}_{vs}{\nabla}^2{C}_{vs}$$

When the drug is transported into the highly vascularised dermis, convective transport of the drug in this layer is not only important but, often, the dominant transport mechanism. Here, vasculature/lymphatic network in the dermis convectively transports drug from the epidermis deeper into the dermis (1, 4). As the capillary loops extend up to the viable epidermal-dermal barrier, convective transport via the capillary loops may be assumed to occur over the full dermal depth. Accordingly, as convection is an order of magnitude faster than diffusion, and thus the transport in the capillary loops can be described by:

$$\frac{\partial {C}_b}{\partial t}=\nabla \left({v}_b{C}_b\right)$$

To solve these governing equations, physiologically feasible boundary conditions must be imposed. An assumed steady-state, i.e. constant, flux (*J*_*sc*_) from the stratum corneum is assumed to enter the top of the viable skin (2) as this applies for most transdermal systems after an initial lag time (19, 20) and the equations defining transport in the stratum corneum when a finite (depleting) dose of product is applied are quite complex (20). A permeability barrier (*P*_*cl*_) was also imposed on the surface of the capillary loop to represent the endothelial lining. Periodic boundary conditions were imposed upon the sides of the computational area. The reason for this assumption is identical computational areas were assumed to be adjacent to the one represented in this model. Finally, a zero flux condition was assumed at the bottom of the region of interest because there was limited variation in solute concentration in the deeper skin layers. The resulting boundary conditions are therefore as follows:

$$\begin{array}{l}J_{sc}=-D_{vs}\frac{\displaystyle\partial C_{vs}}{\displaystyle\partial z}{\left.\right|}_{z=0}\\J_{cl}=k_p\left(C_{vs}-C_b\right)\\\frac{\displaystyle\partial C_{vs}}{\displaystyle\partial y}{\left.\right|}_{y=0}=\frac{\displaystyle\partial C_{vs}}{\displaystyle\partial y}{\left.\right|}_{y=w_{c.a}}\\\frac{\displaystyle\partial C_{vs}}{\displaystyle\partial x}{\left.\right|}_{x=0}=\frac{\displaystyle\partial C_{vs}}{\displaystyle\partial x}{\left.\right|}_{x=l_{c.a}}\\\frac{\displaystyle\partial C_{vs}}{\displaystyle\partial x}{\left.\right|}_{z=800}=0\end{array}$$

In this work, it was assumed that there was no initial concentration of drug in the viable skin or the capillary network, yielding the following initial conditions:

$${C}_{bt=0}=0$$

$${C}_{vst=0}=0$$

Finally, this work extended the well-established approach of using compartments to represent different skin regions, such as the stratum corneum, viable skin and vascular network (21–23) to represent average concentration in and around the subpapillary plexus.

In general, the capillary loops, subpapillary plexus, perforator vessels and the associated arterial input from and venous output into deep tissues must be considered to develop the series of transport equations. Together, this continuous three-dimensional network of vessels that constitute a vascular territory, often called an angiosome, and that have been defined by ink injection visualisation, dissection and radiography (24). In order to address the complexity associated with any attempt to model the full angiosome, with its multiple plexuses, perforator and other vessels, we have limited this analysis to angiosome region including and superficial to the subpapillary plexus, as this region is the main site of pharmacological action for a topical drug product and determines the clearance of a drug from the viable epidermis. Thus, we assume permeation of a drug from the epidermal dermal junction across the avascular dermis region of 50 to 100 μm thick into the dermal papillae as well as into surrounding dermal tissue and, by a process of blood flow convection from the venular branch of the capillary loops arising from the papillae and tissue diffusion, into the dermal plexus. Transport of solutes may also occur from the deeper tissues back to the viable epidermis as occurs in the nutrition of this layer and will occur for drugs redistributing from the systemic circulation into the skin.

In this modelling, plexus transport was represented as occurring through two compartments, representing the venular and arterial components of the plexus. These two compartments interact with each other via capillaries and the model allows for drugs to pass backwards and forwards through capillary loops in a dynamic process. Accordingly, solute transport in the plexus is described by the following expressions:

$${V}_{vp}\frac{d{C}_{vp}}{dt}={A}_{vp}\left({k}_p\left({C_{vs}}_{\mid top\ of\ vp}-{C}_{venule\ plex}\right)+{k}_2\left({C}_{ap}\right)-{k}_1\left({C}_{vp}\right)+\frac{A_{cl}}{A_{vp}}\ {k}_{pcl}\left({C}_{end\ of\ capillary\ loop}\right)\right)$$

$${V}_{ap}\frac{d{C}_{ap}}{dt}={A}_{ap}\left({k}_p\left({C_{vs}}_{\mid bot\ of\ arteriole\ plex}-{C}_{ap}\right)-{k}_e{C}_{ap}-{k}_2\left({C}_{ap}\right)+{k}_1\left({C}_{vp}\right)-\frac{A_{cl}}{A_{ap}}\ {k}_{pcl}\left({C}_{start\ of\ capillary\ loop}\right)\right)$$

where V_vp_ and V_ap_ is the volume of each plexus, *C*_*vp*_ is the concentration in the venule plexus, *C*_*ap*_ is the concentration in the arteriole plexus, *k*_1_ is the rate of drug entering the arteriole plexus from the venule, *k*_2_ is the rate of drug entering the venule plexus from the arteriole, *k*_*pcl*_ is the rate drug enters the venule plexus from the capillary loop and *k*_*e*_ is the rate entering the arteriole section of the capillary loop. Here, *k*_*e*_ = *v*_*b*_*A*_*cl*_ and *A*_*cl*_ is the cross-sectional area of the capillary loop. The cross-sectional area was calculated through the use of COMSOL by taking two integrals; one of the width and one of the length.

A limitation in the above representation is that the subpapillary plexus has more venules than arterioles and, in reality, spaces exist between individual vessels. This spacing has been recognised in this model by the inclusion of pores allowing a drug to diffuse through the subcutaneous plexus without entering the blood vessels. The transport within these pores can be simply expressed as a diffusion process occurring within the dermal tissue that constitute the pores:

$$\frac{\partial {C}_p}{\partial t}={D}_{vs}{\nabla}^2{C}_p$$

Here, as within the plexus compartments, it is assumed that the initial concentration is zero. As a result, the initial condition for the plexus can be satisfied by:

$${C}_{vp}\left(x,y,z,t=0\right)={C}_{ap}\left(x,y,z,t=0\right)=0$$

The above set of equations all combine to give governing equations that describe drug transport in the viable skin and various blood vessels. These equations were solved numerically through the use of COMSOL Multiphysics (25). In order to solve these equations, COMSOL employs a finite element approach. The finite element approach relies heavily on the development of a mesh. In this model, the mesh had to be refined and more granular near the capillary loops as these locations have higher concentration gradient. When the mesh was aptly selected, the concentration and flux inside the viable skin and blood vessels could be predicted.

## Results and Discussion

### Size of Subpapillary Plexus

In practice, the branching of the vasculature network within the subpapillary plexus varies considerably from person to person (11). The consequence is that a change in the branching affects the overall depth of the subpapillary plexus and branches that extend vertically will increase the depth of the plexus. In contrast, branching that extends horizontally will effectively decrease the plexus depth. Accordingly, the impact of subpapillary plexus compartment size on the dermal drug transport of a solute is of some importance.

**Fig. 3:**
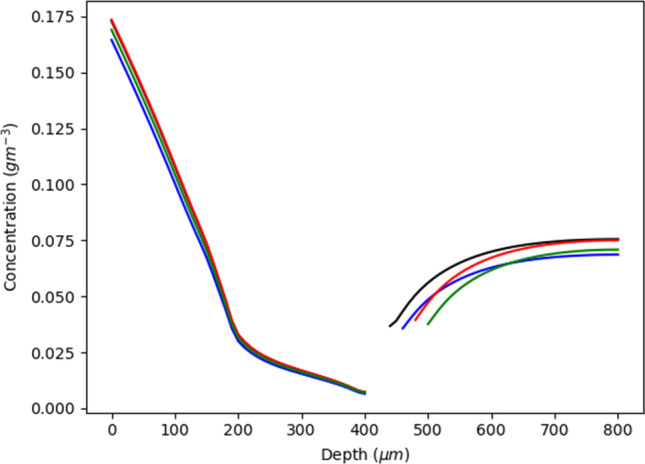
Concentration of a solute at different depths within the viable skin with different sizes for the total plexus. Each of the concentrations is recorded adjacent to the arterial branch of the capillary loop. The blue colour signifies the standard which has a total plexus size of 50*μ*m. The red has total plexus size of 70*μ*m, the green has a sizes of 90*μm*, and the black has a total plexus size of 30*μ*m

Figure 3 shows that drug concentration in the viable skin and dermis decreases until reaching a depth of ~400*μm*, relatively independent of the size of the subpapillary plexus. However, beyond that depth, there is an increase in concentration with depth which is most pronounced for the smallest plexus depth and least for the largest plexus. The result was unexpected in that we had anticipated a lower drug concentration with an increase in subpapillary plexus size. The explanation for this finding is two-fold. A larger subpapillary plexus size leads to an increased amount of drug being unable to diffuse through the ‘viable skin pores’ and instead entering the subpapillary plexus. A larger concentration in the plexus then allows a greater quantity of drug to enter the larger venular blood vessels which enter the deeper dermis and increase concentration. However, when the plexus is small, more of the drug will travel through the pores and into the deeper layers, resulting in an increased drug concentration below the plexus. Another potential reason for the finding may have been the distance to the bottom of the reflective boundary condition at the bottom of the computational area. A smaller distance for larger subpapillary plexus simulations could have resulted in a lesser diffusion distance and thus increased the amount of drug present in deeper layers. The following quadratic regression equation shows the relationship between drug concentration and subpapillary plexus size (*R*^2^ value of 0.94), which defines a minimum drug concentration at the standard plexus size:

$${C}_{plex}=2\times {10}^7{p}_{size}^2-1990.4{p}_{size}+0.0454$$

where *C*_*plex*_ is the average concentration in the plexus and *p*_*size*_ is the size of the plexus.

The equation is most reliable for the smaller values of plexus depth where the plexus pores dictate transport and enhance drug concentrations below the subpapillary plexus. On the other hand, larger plexus sizes facilitate convective capillary solute convection and results in more drug being retained in the subpapillary plexus.

### Depth of Subpapillary Plexus

**Fig. 4:**
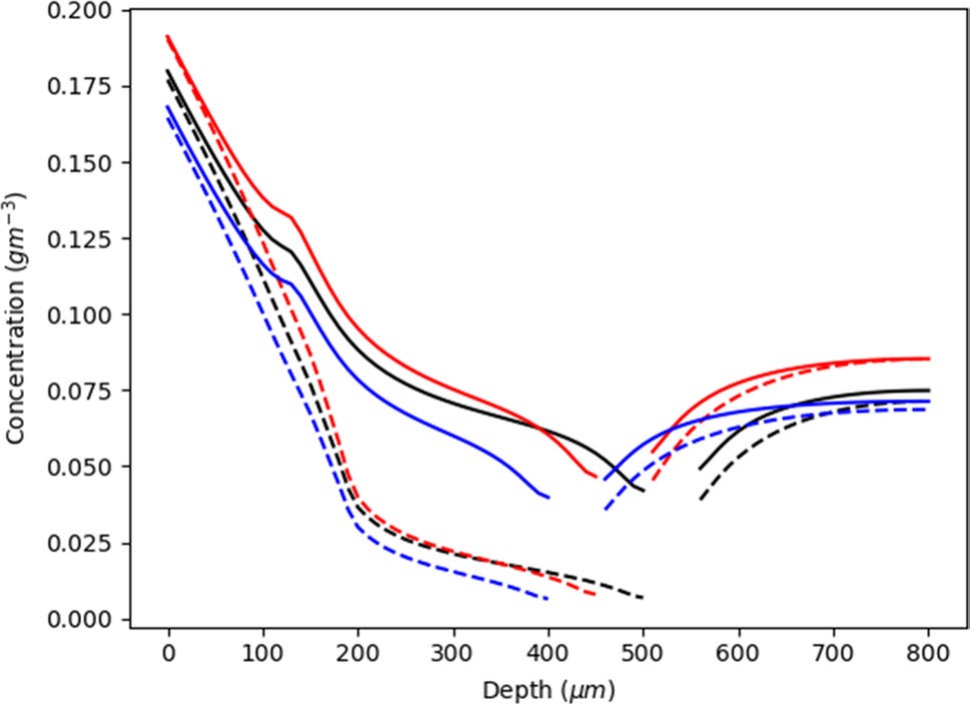
The figure shows the concentration at different depths within the viable epidermis and dermis. The gaps in concentration are related to the area of the subpapillary plexus. The dotted lines represent the concentration near the arteriole, while the solid lines show the concentration near the venule. The red colour represents a subpapillary plexus depth of 450*μ*m, the blue colour is the standard depth of 400*μ*m, and the black colour is the deepest plexus with a depth of 500*μ*m}

The depth of subpapillary plexus can vary greatly as a result of variations in physiology, with microneedle application significantly decreasing the depth to 240*μm* (26) and temperature induced capillary recruitment increasing plexus depth. As plexus drug concentrations are also dependent on whether the concentration being measured is adjacent to either the venular and arteriole sections of the capillary loop (1), location within the plexus was considered in this analysis. Figure 4 shows the resulting profiles. It is evident that significant concentration differences exist between regions near to the arteriole and venules in both the upper viable skin and dermis, with the highest concentrations being found near the venule. This difference becomes more pronounced with increasing dermal depth and highest when the depth of the plexus is 450*μ*m. The arteriole - venule concentration gradient appears to disappear in the region below 450*μ*m, with the drug concentration in the lower regions of the dermis being greater than above the plexus. This higher concentration probably arises from capillary loops transporting more drug into the lower dermis.

A quadratic regression analysis of multiple simulations shows that plexus drug concentration *C*_*plex*_ is related to *h*_*plex*_ with an *R*^2^ value of 0.95.

$${C}_{plex}=-199723{h}_{plex}^2+182.39{h}_{plex}-0.0387$$

where *h*_*plex*_ is the depth of the plexus. As in Fig. 2, the highest drug concentration occurs at a dermis depth of ~450*μ*m. Higher concentrations are seen at lesser depths, where smaller capillary length led to less drug entering the capillary loops and being transported into the subpapillary plexus and larger blood vessels. In contrast, in very deep plexuses, the plexus is a long way from the top of the skin so that the amount of drug entering the top of the compartment is reduced, as drug back diffusing into the viable skin from the capillary loops.

### Velocity of the Blood

**Fig. 5:**
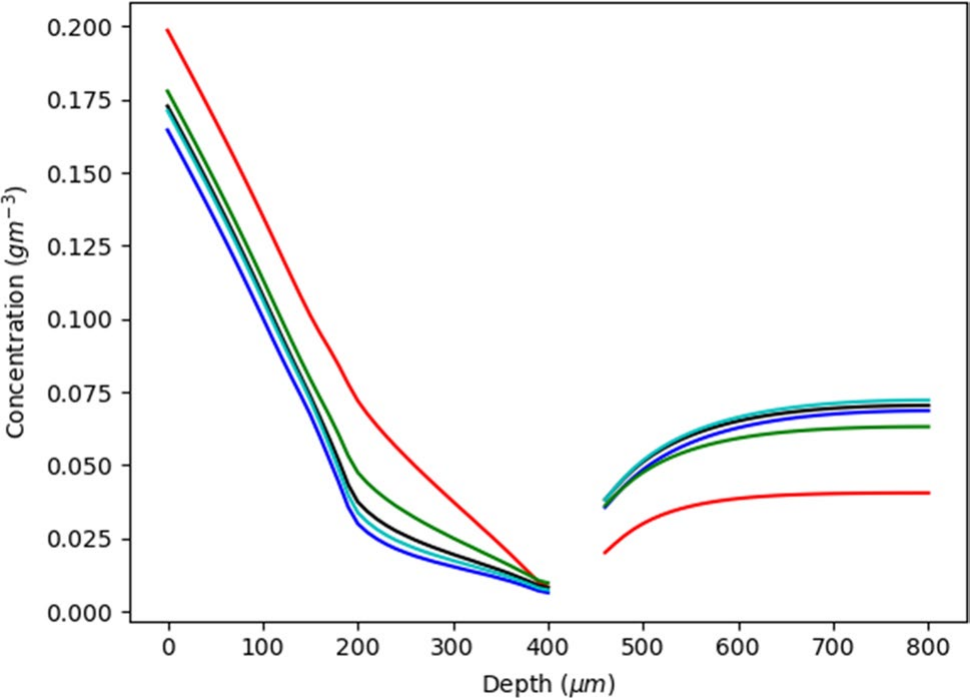
The concentration of solute present at each depth when different velocities of blood in the capillary loops were imposed. The concentrations were all taken adjacent to the arteriole. The blue colour was the standard and had the velocity stated in the method (0.65 ± 0.3 *mm*/*s*). The red line considers when the velocity was tenfold smaller than the standard velocity. Meanwhile, the green line considered ¼ of the standard velocity, the black line implemented ½ of the standard velocity, and the cyan line used ¾ of the standard velocity

The third parameter studied in this work was blood velocity, which has been reported to have a skin value of 0.65 ± 0.3 *mm*/*s* (12), with an associated large variability (1). Figure 5 shows a similar drug concentration with distance profile as shown for Figs. 3 and 4.

In general, above the subpapillary plexus, a lower blood velocity is associated with a higher drug concentration of the solute. Importantly, at the lowest blood velocities, blood convection may have a limited effect and tissue diffusion becomes a dominant means of transport and maintaining drug concentrations within vessels. A regression analysis of the data from multiple simulations resulted in the following exponential decline in drug concentration in the plexus (*R*^2^ value of 0.97).

$${C}_{plex}=0.008{e}^{-2038{v}_b}$$

In contrast, in the region below the subpapillary plexus the opposite occurs in that blood concentration decreases as the velocity of the blood decreases and is especially profound when there is a significant decrease in velocity.

### Density of Blood Vessels

The fourth parameter considered here is the density of blood vessels within the subpapillary plexus. In this case, density is represented by introducing avascular diffusion pores that penetrate through the subpapillary plexus compartment. We acknowledge that this approach arose from a recent blog reporting that microneedle arrays significantly changed the density of blood vessels in the dermis (26). The study showed that microneedles decreased the depth of the plexus from 430μm to 241μm and increased the density of blood vessels in the plexus from 9% to 40%.

**Fig. 6:**
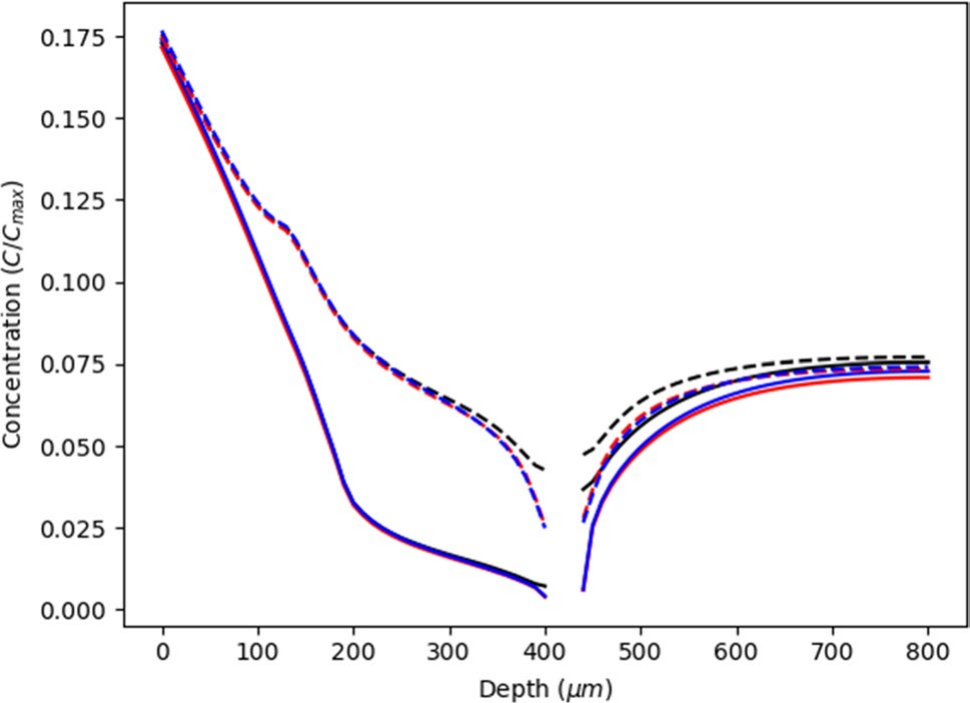
The figure shows the concentration when the density of the pores in the subpapillary plexus was changed. The dashed lines represent the concentration adjacent to the venular branch of the capillary loop while the solid line represents the concentration near the arteriole branch. The black colour represents the standard density of 11%. The red colour represents a pore density of 19% while the blue represents a pore density of 25%

Figure 6 shows the impact the blood vessel density in the subpapillary plexus drug concentration within the viable skin. It is evident that relative to the physiological measures studied previously, blood vessel density has a somewhat marginal impact. Largest concentration differences are seen between near the venule and the arteriole at a depth range of 350-400μm, with a higher concentration near the venule also being found below the subpapillary plexus. As shown earlier in Figs. 3, 4 and 5, all concentrations increase at deeper dermis locations.

The best model to describe the concentration in the subpapillary plexus data obtained here is a simple regression:

$${C}_{plex}=0.0056{p}_d+0.002$$

where *p*_*d*_ is the density of pores in the subpapillary plexus. This model is simply explained by the pores acting as a drug concentration supply so that more pores means a higher concentration.

### Comparison of Previous Capillary Models

**Fig. 7:**
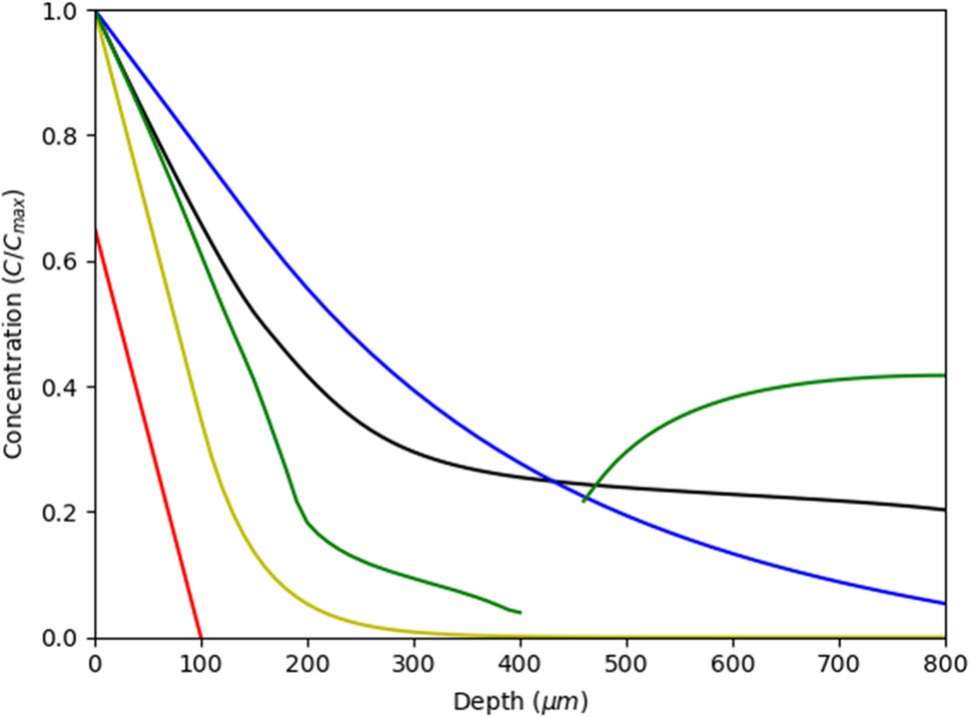
A comparison of mathematical models which aim to explain the impact of the blood vessels. The green line represents the concentration profile for the subpapillary plexus model introduced in this paper. The black line represents the enhanced capillary model in [1], while the yellow line represents the first capillary model in [2]. The simple linear model is described by the red colour while the previously used distributed-elimination model is shown in blue

The models described here add to those used previously such as the homogenous membrane model but are a more realistic representation of transport within the skin. The variations in predicted drug concentration profiles of the available models relative to that described here shown in Fig. 7, with the strengths and limitations of each model listed in Table I.

**Table I:** Comparison of Mathematical Models that Aim to Describe Drug Transport in the Dermis

Mathematical Model	Strengths	Limitations
Simple homogenous model	• Easy to use model • Provides a reasonable estimation for the superficial viable epidermis region.	• Lack of applicability when looking at dermal regions. • Much less accurate with larger membranes. • Dependent on the location of the sink condition.
Distributed-elimination model (27)	• Reasonable easy to use. • Can give a good estimation of the viable epidermis as well as superficial dermis.	• Does not accurately predict deeper dermal concentrations. • Dependent on using an elimination rate that is hard to determine to estimate the impact of blood vessels. • The transient profile is much slower than what is seen in experimental results.
Convection dispersion diffusion - elimination model (3)	• Improved model from distributed elimination model. • More accurate representation of transport by incorporating diffusion and convection. • Better representation of transient profile, although it is still accurately depict experimental results.	• Developing a dispersion coefficient is often from experimental data for each profile. • Difficult to estimate what impact convection has. • Does not account for permeation from capillary loops back into the skin
Simplified capillary model (2)	• Is able to model the capillary loops explicitly and discuss the effect of changing parameters. • Provides a more accurate representation of deeper dermal skin layers than the above methods. • Can be used to estimate position to place a sink condition for the homogenous model.	• Does not account for permeation back into the skin from the capillary loops. • Does not consider plateauing of the concentration at deeper dermal regions. • Transient profile is slower than experimental results and matches distributed elimination model. • Position of maximum and minimum less concentration at each depth may not be accurate (between capillary loops).
Enhanced capillary model which incorporates convection within capillary loops (1)	• Accounts for convective transport of blood vessels. • Concentration profile of deeper dermis is much more accurate. • Accounts for a plateau in concentration-depth profile. • Much more accurate transient profile.	• Large computational time due to sensitivity of the mesh. • Extended capillary loops oversimplify the deeper dermis vasculature.
Subpapillary plexus model	• First model to consider subpapillary plexus. • Easier to use model for subpapillary plexus concentration since it uses compartments instead of mapping all vessels. • Accounts for drug transport between blood vessels. • Considers the case where concentration can re-enter capillary loops and be redistributed to upper dermis.	• Long computational time. • Does not consider capillary branching in the plexus. • Subpapillary plexus is roughened by two compartments. • Model needs to be further developed to become more accurate at estimating deeper dermal concentration, large venules and arterioles need to be expanded upon.

The homogenous model predicts that drug concentration decreases linearly with dermis depth (Fig. 7), which coincides with actual viable concentrations at early but not later times, especially in the deeper dermis. The refined convection dispersion diffusion -diffusion model more accurately predicts deeper dermal concentration declines in an exponential manner but is limited to regions deeper than 200μm as the plexus is ignored and, as a result, the predicted drug concentration decreases more rapidly with distance than is observed. A similar issue arises with the first capillary model (2) where a similar trend and where a sink condition applied to capillary loops ensures a negative exponential decay. Of these latter two models, the convection dispersion diffusion - elimination model has a much lower gradient as it recognises deeper tissue transport involves both blood convection and tissue diffusion. Incorporates a dispersion coefficient, which is a coefficient that estimates both blood convection and tissue diffusion.

A more advanced capillary model exhibits a non-exponential decay whereby there is a plateauing of concentration below the capillary loops (1) and is supported by experimental data (28, 29). Finally, the subpapillary plexus model presented in this paper predicts an even more profound increase in deeper dermal concentrations. In this model, the concentration below the subpapillary plexus is approximately 30% of that at the top of the viable skin.

Each model displayed has a functionality which can be useful in a quality-by-design approach to skin delivery to deeper tissues. The simple linear model provides a reasonable estimate of concentration in the upper parts of the viable skin and is a quick and easy tool that requires limited mathematical ability or software capability. The final equations for concentration obtained from the distributed elimination model and dispersion elimination model are also quick and relatively easy models to use, provided a diffusion or dispersion coefficient can be identified. For more accurate representations of maximum, minimum and transient concentration profiles, it is more important to explicitly model capillaries in the viable skin. The first capillary model, which applied sink conditions inside the bloodstream, is a good steppingstone towards starting to account for many of the convective transport issues that smaller molecular weighted drugs might experience. The second convective transport model is a much more realistic expression of convective transport and should be employed for people concerned about impacts on maximum concentration or transient concentration. In this model, the time to reach steady state is many-fold faster than any of the other models and matches what has been seen in previous experimental data. Finally, the more recent subpapillary plexus model further investigates the impact of the blood vessels. The model accounts for capillary branching in the plexus, as well as the possibility of repermeation to upper layer, thus giving it the most physiological relevancy. Therefore, it gives the most accurate spatial-based concentration predictions and has the best representation of convective transport in the viable skin. However, it is quite complicated to design computationally and mathematically. The real benefits of the model could be further realised in future work through future enhancement and simulation, where the complicated model can be exported into a much more user-friendly device. In the future, the more complicated models could be used to develop simpler ‘single use’ expressions of permeation that would be more accurate and faster than what other models are able to predict. The maximum and minimum concentration work could also be generalised into simpler equations which could provide simple estimations that could be employed for toxicological and therapeutic reasons. The more complicated models may also provide a realistic simulation of how a drug will spread through the skin before invitro experimentation, which could limit the toxicological impact on testing subjects.

## Conclusion

This work has introduced a new dermal transport model useful for describing drug concentration in the dermis after a topical application or exposure. It has shown that drug concentrations are related to location proximity to arteriole and venule components of the dermal plexus and dermis depth. In particular it has used COMSOL and physiologically based pharmacokinetic modelling to define the impact on dermal concentrations of subpapillary plexus size, depth, blood velocity and density of subpapillary plexus vessels. The resulting concentration - depth profiles obtained are more consistent with the available experimental data than any previous models used for this purpose.

## Abbreviations

*D*_*vs*_: Diffusion coefficient in the viable skin (m^2^/s)
*D*_*p*_: Diffusion coefficient in the viable skin (m^2^/s)
*h*_*vs*_: Height of the viable skin for the model (*μm*)
*l*_*cl*_: Distance between capillary loops (*μm*)
*l*_*arch*_: Arch distance for capillary loop (*μm*)
*l*_*c*. *a*_: Length of the computational area (*μm*)
*w*_*c*. *a*_: Width of the computational area (*μm*)
*k*_*cl*_: Permeability of the capillary loops (*m*/s)
*C*_*p*_: Concentration between vessels in the plexus (*g*/*m*^3^)
*C*_*b*_: Concentration in the capillaries (*g*/*m*^3^)
*C*_*vs*_: Concentration in the viable skin (*g*/*m*^3^)
*v*_*b*_: Velocity of blood in the capillaries (*mm*/*s*)
*J*_*cl*_: Flux entering the capillary loops from the viable skin (*gm*^−2^ *s*^−1^)
*J*_*in*_: Flux entering the capillary loops from the arteriole plexus (*gm*^−2^ *s*^−1^)
*J*_*sc*_: Flux entering the viable skin from the stratum corneum (*gm*^−2^ *s*^−1^)
*J*_*out*_: Flux exiting the capillary loops and entering the venule plexus (*gm*^−2^ *s*^−1^)
*p*_*size*_: Size of the 2 plexuses combines (*μm*)
*P*_*d*_: Ratio of Pores in plexus to blood vessels (1)
*h*_*plex*_: Depth of each plexus (*μm*)
*C*_*plex*_: Average concentration in each plexus (*g*/*m*^3^)
*C*_*ap*_: Concentration in the arteriole plexus (*g*/*m*^3^)
*C*_*vp*_: Concentration in the venule plexus (*g*/*m*^3^)
V_ap_: Volume of the arteriole plexus (m^3^)
V_vp_: Volume of the venule plexus (m^3^)
*A*_ap_: Area at top of the arteriole plexus (excl capillary loops) (m^2^)
*A*_vp_: Area at top of the venule plexus (excl capillary loops) (m^2^)
*k*_*p*_: Permeability between plexus and viable skin (*m*/*s*)
*k*_1_: Permeability from venule plexus to arteriole (*m*/*s*)
*k*_2_: Permeability from arteriole plexus to venule (*m*/*s*)
*k*_*pcl*_: Permeability from arteriole plexus to capillary loop (*m*/*s*)
